# An unusual cause of hepato-biliary disease in an immunocompromised patient

**DOI:** 10.1099/acmi.0.000049

**Published:** 2019-08-07

**Authors:** Alison Johnson, Sajal Gupta, Simon Meyrick, Angharad P. Davies, Rachel Chalmers

**Affiliations:** ^1^ Wye Valley NHS Trust, County Hospital, Union Walk, Hereford, Herefordshire, HR1 2ER, UK; ^2^ Cryptosporidium Reference Unit, Public Health Wales Microbiology ABM, Singleton Hospital, Sketty, Swansea, SA2 8QA, UK

**Keywords:** cryptosporidium, hepato-biliary, nitazoxanide

## Case summary

A 14-year-old girl presented with diarrhoea, vomiting and abdominal pain after a holiday in the UK. She had been on tacrolimus 5mg bd for focal glomerulosclerosis since the age of 13 years. She recovered, but presented again after 19 days with further abdominal pain and abnormal liver function [aspartate transaminase, 451 U l^−1^ (normal range 0–32 U l^−1^); alanine transaminase, 267 IU l^−1^ (normal range 0–33 IU l^−1^); gamma-glutamyl transferase, 115 U l^−1^ (normal range 1–40 U l^−1^); and lactate dehydrogenase, 853 U l^−1^ (normal range 240–480 U l^−1^)]. An abdominal ultrasound scan demonstrated a thickened gall bladder, but otherwise normal liver and bile ducts.

**QUESTION**

Which common gastrointestinal pathogen causes hepato-biliary involvement in patients who are immunocompromised?

**ANSWER OPTIONS**

- *
Salmonella
*
- *Cryptosporidium*
- *
Escherichia coli* O157
- *
Campylobacter
*

## Discussion

Answer: (b) *Cryptosporidium*. In this patient, the diagnosis of *Cryptosporidium* was made by stool microscopy. Cultures for *Salmonella *, *Shigella *, *Escherichia* *coli* O157 and *Campylobacter* were negative. *Cryptosporidium* is known to cause hepato-biliary involvement in immunocompromised patients. It is most frequently associated with severe T-cell deficiency, such as is seen in human immunodeficiency virus infection, with CD4 counts lower than 200; in haematological malignancies; and in patients with primary T-cell deficiencies [1]. This case demonstrates that this complication can also occur in patients with relatively mild immunosuppression. The patient made a good recovery on nitazoxanide 500 mg bd for 3 days. Clinicians should be aware that cryptosporidium can cause biliary involvement in immunocompromised patients and, conversely, cryptosporidium should be considered and tested for in immunocompromised patients with deranged liver function tests and/or biliary abnormalities.
